# Transfusion‐related acute lung injury under general anesthesia successfully treated with extracorporeal membrane oxygenation: A case report

**DOI:** 10.1002/ccr3.7386

**Published:** 2023-05-20

**Authors:** Yusuke Ishida, Koichi Nakazawa, Toshio Itabashi, Mikiko Tomino

**Affiliations:** ^1^ Department of Anesthesiology Tokyo Medical University Tokyo Japan

**Keywords:** platelets, transfusion‐related acute lung injury, veno‐venous extracorporeal membrane oxygenation

## Abstract

Transfusion‐related acute lung injury (TRALI) is a serious complication of blood transfusion and can also develop severe hypoxemia. In TRALI cases with difficult blood oxygenation on mechanical ventilation support, temporary veno‐venous extracorporeal membrane oxygenation support appears to maintain oxygen levels.

## 1 BACKGROUND

Transfusion‐related acute lung injury (TRALI) is characterized clinically by the onset of acute respiratory distress syndrome (ARDS) within 6 h of initiation of blood transfusion with difficulty breathing, hypoxemia, hypotension, and bilateral pulmonary edema.^1^ TRALI is a major cause of blood transfusion‐related death. The differential diagnosis of TRALI includes heart failure and pneumonia. TRALI occurs in the perioperative period in 1.3%–1.4% of patients who receive a blood transfusion.^2^ Though approximately 80% of patients recover from TRALI within 2–3 days, the reported mortality rate is 13%–18%.^3^, ^4^ The case of a patient who developed TRALI after intraoperative platelet transfusion in whom veno‐venous extracorporeal membrane oxygenation (VV‐ECMO) support for severe and refractory hypoxemia was effective for life‐saving is presented. The patient's written, informed consent for publication of this case report was obtained.

## 2 CASE PRESENTATION

**FIGURE 1:**
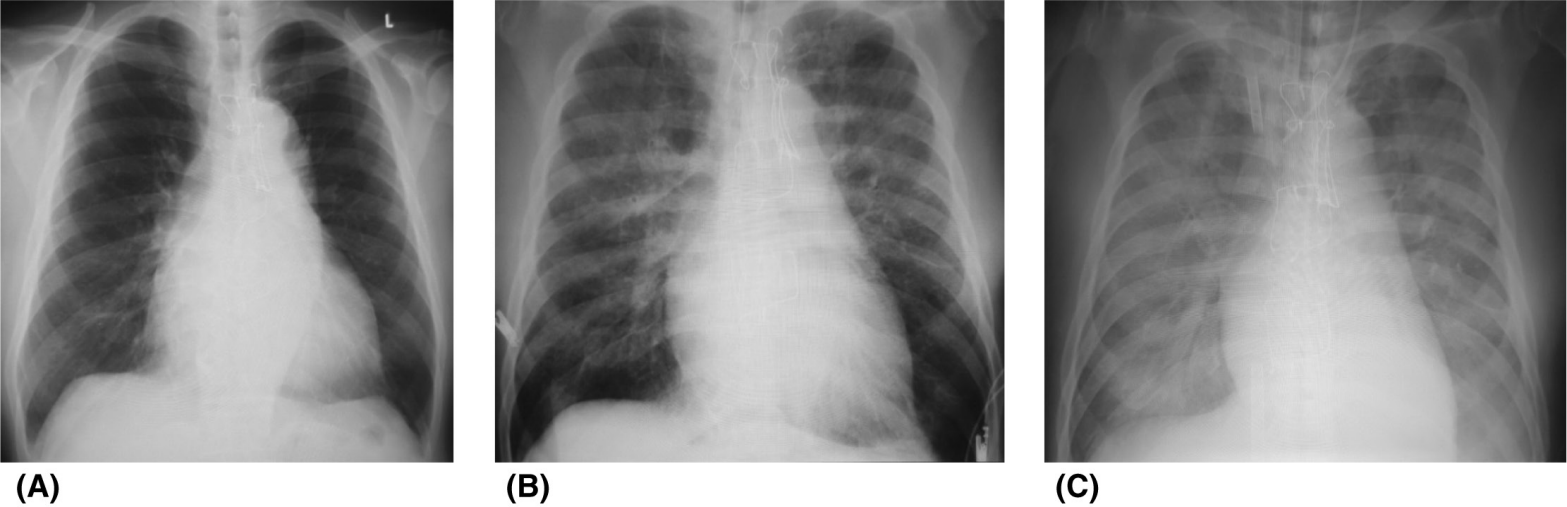
Chest X‐ray. (A). Preoperative chest X‐ray. Chest X‐ray shows clear lung fields. (B). First postoperative chest X‐ray. Postoperative chest X‐ray shows bilateral pulmonary edema. (C). Chest X‐ray after reoperation. Chest X‐ray at the beginning of VV‐ECMO support shows infiltration in the entire lung field.

A 79‐year‐old man (height, 169 cm; weight, 79 kg) was scheduled for total cystectomy and ileal conduit diversion for bladder cancer. The patient had a history of acute myeloid leukemia and achieved remission by chemotherapy 9 years earlier. He was diagnosed with a thoracic aortic aneurysm and myocardial infarction, and underwent total arch replacement and coronary artery bypass grafting 3 years earlier. His blood pressure, heart rate, and peripheral blood oxygen saturation (SpO_2_) while breathing room air were 110/70 mmHg, 90 bpm, and 98%, respectively. On chest X‐ray, the cardiothoracic ratio (CTR) was 58% with normal lung fields (Figure 1A). Transthoracic echocardiography showed normal cardiac function with left ventricular ejection fraction (LVEF) of 50%. The complete blood cell count showed anemia and thrombocytopenia (RBC 2.83 × 10^6^/μL, hemoglobin 8.9 g/dL, hematocrit 26.7%, and platelets 1.0 × 10^5^/μL). He was scheduled to receive intraoperative platelet transfusion.

For anesthesia induction, he received intravenous midazolam 7 mg and remifentanil 0.2 μg/kg/min, and endotracheal intubation was carried out with rocuronium 50 mg as a muscle relaxant. Anesthesia status was maintained using 4% desflurane and remifentanil 0.1–0.2 μg/kg/min. When his blood pressure dropped, ephedrine and/or phenylephrine was administered as needed. Standard intraoperative monitoring and invasive blood pressure measurement were carried out. Platelet transfusion was initiated at the beginning of the surgery, and the blood oxygenation level of the patient gradually decreased 40 min after the transfusion, with SpO_2_ of 92%–95% at fraction of inspired oxygen (FiO_2_) of 0.5, and a large amount of foamy sputum was suctioned from the endotracheal tube. The patient became hypotensive, and noradrenaline administration was initiated. The patient was transfused with packed red blood cells (560 mL), platelets (400 mL), and 5% albumin (1000 mL). The total amount of fluid infusion was 5100 mL; total blood loss was 1552 mL, and total urine volume was 100 mL. Total operation time was 3 h 59 min, and total anesthesia time was 5 h 18 min. The postoperative chest X‐ray showed bilateral pulmonary edema (Figure 1B). The patient showed a poor oxygenation level with a PaO_2_/FiO_2_ (P/F) of 70.1 and was transferred to the intensive care unit (ICU) under sedation and mechanical ventilation. After transfer to the ICU, bleeding from the drainage tube continued, and anemia progressed. Therefore, he was scheduled for emergency hemostasis in the operating room 1 h after admission to the ICU.

Anesthesia was maintained using desflurane and remifentanil, and continuous administration of sivelestat was added. Steroid was also administered to correct increased vascular permeability. During the operation, foamy secretions were continuously produced from the endotracheal tube and removed using a bronchofiberscope and a suction tube. A recruitment maneuver was also manually carried out whenever necessary. TRALI was suspected from the clinical course. After surgical hemostasis, blood gas analysis at FiO_2_ 1.0 showed pH 7.06, PaCO_2_ 96.1 mmHg, PaO_2_ 67.9 mmHg, and HCO_3_–26.1 mmol/L, indicating severe hypoxemia and hypercapnia. VV‐ECMO support was then started.

His chest X‐ray at the beginning of VV‐ECMO support showed infiltration in the entire lung field (Figure 1C). He received continuous treatment with steroid and sivelestat. After VV‐ECMO support was started, hypoxemia and hypercapnia improved markedly. Under VV‐ECMO support, mechanical ventilation with driving pressure at 10 cmH_2_O, PEEP at 10 cmH_2_O, and FiO_2_ at 0.4 was provided. His lung condition improved gradually, and foamy secretions decreased from postoperative day (POD) 2. The chest X‐ray image improved markedly. His further clinical course was good, and he was weaned off VV‐ECMO on POD 6 and mechanical ventilation on POD 7. He was then discharged from the ICU. Antibody testing of donor serum was performed for TRALI diagnosis, and the anti‐HLA Class II antibody was positive, and a positive cross reaction to the patient's white blood cells was confirmed.

## 3 DISCUSSION

The present patient showed reduced blood oxygenation levels after platelet transfusion and foamy endotracheal secretions. Therefore, TRALI was strongly suspected during the surgery. TRALI is characterized by hypoxemia within 6 h after blood transfusion and bilateral lung infiltrations on chest X‐ray.^5^ There are two types of TRALI (types I and II). The present case appeared to be TRALI type I based on his symptoms and clinical course.^6^ Repeated suction of endotracheal serous discharge and the recruitment maneuver at FiO_2_ 1.0 was performed during surgery to ensure oxygenation. The present case of TRALI had a good clinical course with systemic organ support by VV‐ECMO in the ICU.

Two underlying mechanisms of TRALI are considered, immunological and non‐immunological mechanisms.^6^ As the immunological mechanism, donor or recipient‐derived antibodies against human leukocyte antigens (HLAs) and human neutrophil antigens (HNAs) have antigen–antibody reactions and activate complement and white blood cells, which may increase pulmonary vascular permeability resulting in non‐cardiogenic pulmonary edema.^7^ As a non‐immunological mechanism, pulmonary vascular endothelial cell injury is evoked by certain causes, and cytokines and active lipids within transfused blood may be associated with the development of non‐cardiac edema.^8^ However, the true pathogenesis has not yet been elucidated. In the present case, anti‐HLA Class‐II antibody within transfused donor serum may have reacted with recipient white blood cells, which triggered the onset of TRALI. The previous hematological malignancy and heart disease were thought to be recipient risks for TRALI, and platelet product transfusion and anti‐HLA/HNA antibodies were donor risks for TRALI.^5^ Though the present case showed TRALI, the possibility that the large volumes of transfusion and fluid therapy during the operation caused an excess volume load postoperatively could not be ruled out.

There are no established effective medications and/or specific treatments for TRALI, and only supportive and symptomatic therapies are currently given. Although there is a report that steroid was used for TRALI treatment,^9^ the effectiveness of steroid for TRALI has not yet been proven.^10^ We are not aware of any previous reports that VV‐ECMO was used for TRALI treatment. Lee et al. reported a case of hypoxemia and circulatory collapse after transfusions of red blood cells, platelets, and fresh‐frozen plasma that was rescued by venoarterial‐ECMO (VA‐ECMO) support.^11^ Nouraei et al. reported a 4‐year‐old case of TRALI caused by transfusion of fresh‐frozen plasma during open‐heart surgery with cardiopulmonary bypass that required postoperative VA‐ECMO support for 15 h.^12^ Kuroda et al. reported a TRALI case after transfusion of packed red blood cells and fresh‐frozen plasma at hepatectomy.^13^ Their case received VV‐ECMO due to a reduced P/F of 76 on the second ICU day. Blood oxygenation improved within a short time, and the patient was successfully weaned off VV‐ECMO on the 4th ICU day and mechanical ventilation on the 8th ICU day. TRALI is a reversible disturbance in which many cases recover within 24–48 h after onset. However, some cases deteriorate and require prolonged treatment.^14^ In cases whose status deteriorates rapidly with life‐threatening hypoxemia and poor respiratory status, VV‐ECMO support appears to be effective. A case of TRALI that developed during surgery in which VV‐ECMO was effective was described.

The present case of TRALI was triggered by intraoperative platelet transfusion and required VV‐ECMO support. Further studies are necessary to elucidate the underlying mechanisms of TRALI, and technical progress in antibody detection methods is needed to prevent TRALI and develop new treatments. Results from large‐scale clinical studies of the intraoperative management of TRALI are needed to improve the clinical outcome of this pathological condition.

## AUTHOR CONTRIBUTIONS

**Yusuke Ishida:** Conceptualization; writing – original draft. **Koichi Nakazawa:** Conceptualization; writing – review and editing. **Toshio Itabashi:** Conceptualization; writing – original draft. **Mikiko Tomino:** Conceptualization; writing – review and editing.

## FUNDING INFORMATION

None.

## CONFLICT OF INTEREST STATEMENT

The authors declare that they have no competing interests associated with this manuscript.

## ETHICS STATEMENT

Not applicable.

## CONSENT STATEMENT

Written, informed consent was obtained from the patient for publication of this case report and the accompanying images.

## Data Availability

The data that support the findings of this study are available from the corresponding author upon reasonable request.
